# Parameter Estimation of Micro-Motion Targets for High-Resolution-Range Radar Using Online Measured Reference

**DOI:** 10.3390/s18092773

**Published:** 2018-08-23

**Authors:** Yu Xing, Peng You, Shaowei Yong

**Affiliations:** Department of Electronic Science, National University of Defense Technology, Changsha 410000, Hunan, China; ynudt@126.com (Y.X.); ysw_nudt@vip.126.com (S.Y.)

**Keywords:** rotational motion, translational motion, HRRP, Wave Transform, parameter estimation

## Abstract

Micro-motion dynamics produce Micro-range (m-R) signatures which are important features for target classification and recognition, provided that the range resolution of radar signal is high enough. However, dechirping the echo with reference measured by narrow bandwidth radar would generate the residual translational motion, which exhibits as random shifts of envelopes of range profiles. The residual translational motion would destroy the periodicity of m-R signatures and make a challenge to estimate rotational parameter. In this work, we proposed an efficient high-resolution range profile (HRRP)-based method to estimate rotational parameter, in which online measured reference distances are used to dechirp the radar raw echo. Firstly, the deduction for the modified first conditional comment of range profiles (MFCMRP) is introduced in detail, and the MFCMRP contain periodic and random components when dechirped by measured reference, corresponding to the rotational motion and the reference measured errors compared with actual reference. Secondly, the Wavelet Transform (WT) is utilized to separate the measured errors from the MFCMRP. The estimations of measured errors are used to compensate the MFCMRP, and then autocorrelation is performed on the estimated periodic component to obtain the estimation of rotational period. Lastly, the rotational amplitudes and phases are achieved by inverse Radon transform (IRT) of the compensated HRRP. The effectiveness of the proposed method in this paper is verified by synthetic data and measured radar data.

## 1. Introduction

Micro-motion [1] refers to a minor motion part in a whole target, and its characteristics of periodic motion could be utilized to estimate the parameters of micro-motion for target classification and recognition [2,3]. Micro-motion mainly consists of three types, such as rotation, procession, and vibration [4]. Rotation is a popular micro-motion widely seen in rotational blades of helicopters [5], rotational antenna of the navy, and so on. Because of noncooperation, moving targets contain translational motion and rotational motion. Translational motion degenerates the parameter estimation via destroying the periodicity of micro-Range (m-R) and micro-Doppler (m-D) signatures. Thus, estimation and compensation of the translational motion is essential before the parameter estimation of rotational motion.

The conventional method for translational motion compensation in a micro-motion target mainly utilizes the m-D signatures of rotational [5], processional [6], and vibrating motion [4]. An effective orthogonal matching pursuit based [5] method was proposed to estimate translational motion by selecting the optimal velocity atom among the velocity dictionary. However, the order of translational motion is limited to two and the estimation accuracy would be affected by the precision of dictionary atoms. Zhang et al proposed a method employing circular correlation coefficients of time-frequency representation to estimate the period of micro-motion [7]. However, no way was provided to estimate other parameters such as micro-motion amplitude, phase, and so on. The amplitude and phase of micro-motion can be utilized to estimate the length and the relative position of the rotational blade, which contribute to target recognition as target motion character. Zhou [8] proposed an efficient way to extract micro-Doppler curves and estimate micro-motion parameters depending on the occlusion effect, but it didn’t consider the translational interference. There is an effective method proposed by Jung [9] to estimate processional parameters based on range alignment and 2D entropy of the micro-Doppler image respectively, but the computation of that is large and SNR requirement is high.

The sparse representation method is necessary for parameter estimation [10,11] and image reconstruction [12] of a rotating target in the case of sparse-driven data. The parametric methods motivated in [10,11] need certain iterations, but the convergence of the iteration process is not strictly proved. Nguyen [12] proposed a method to focus the blurred image of a rotating target based on sparse-driven data, but this method consider the data after translational compensation.

The methods referred to above all use the m-D signatures, and they are effective to estimate the micro-motion parameters when neglecting some factors. For example, the m-D signatures are easily limited by pulse repetition frequency (PRF) and generate doppler ambiguity, that is, doppler curves aliasing [7]. Zhang proposed an efficient method to estimate the processional period via the circular correlation coefficients of time-frequency representation. However, no way was provided to obtain other parameters such as processional amplitudes, and this method also has high signal-to-noise ratio (SNR) requirements [7]. 

The methods proposed before mainly modeled the translational motion as a polynomial, which required that the translational motion is slight to be displayed in the imaging scope. What’s more, the translational motions motivated in these papers are limited in a short range. If the translational range is large, it makes the range of image extension large and the extension hard to determine. One solution to counter the effect of large image extension is to dechirp the radar echo by online measured reference, and it utilizes the entropy of m-R signatures to estimate the micro-motion period of a cone-shaped target. However, the first conditional moment of original range profiles is sensitive to noise, which would make the initial estimation of translational motion useless and the period estimation wrong [6].

In actual radar imaging applications, the measured reference is usually utilized to determine the scope of imaging, but the reference measured by narrowband radar signal always interfered with measured error, which results from the variance of attitude in micro-motion. For a slow-varying attitude target, the relative distances between different scattering points are unchanged, and the maneuverability of the target would be solved by the correlation of the envelopes of range profiles. However, for the micro-motion target, there isn’t a stable reference because the attitude is varying heavily and periodically, and the ranging errors from narrowband radar signal must also be considered and compensated.

In this paper, we propose a novel HRRP-based method to estimate the rotational parameter in the presence of translational motion, where the reference distance is measured by narrowband radar signal in real-time. The modified first conditional moment of range profiles (MFCMRP) is introduced to express the variance of HRRPs, and then the Wavelet Transform (WT) is utilized to separate the random item from the periodic item. For the two components in the MFCMRP, the former is used to compensate the contaminated HRRPs for the parameter estimation of rotational motion through inverse radon transform (IRT) [13], and the latter is utilized to estimate the rotational period via autocorrelation. Since online measured reference is used to dechirp radar echo, large translational motion would not make a long range HRRP or introduce additional noise. Compared to the first conditional moment of original range profiles [6], the MFCMRP is more robust to noise and more precise to describe the translational motion of a rotating target. Moreover, the effectiveness of the proposed method is verified by the measured data in an absorbing room.

## 2. Micro-Range Signature

Without loss of generality, the point-scatter model is usually constructed to describe the radar signal scattered by a target. In this paper, the linear-frequency modulated (LFM) signal is utilized to acquire the m-R signature. Dechirping the echo of target with a reference signal and compensating the residual video phase, we can obtain HRRPs of the rotational target [6].

(1) $$H\left(r,t_{m}\right)=\sum_{k=1}^{K}\sigma_{k}\left(t_{m}\right)T_{p}\sin c\left\{\frac{2B}{c}\left(r-\left(r_{k}\left(t_{m}\right)-r_{ref}\right)\right)\right\}\cdot \exp\left[-j\frac{4\pi f_{c}}{c}\left(r_{k}\left(t_{m}\right)-r_{ref}\right)\right]$$

where $\left(r,t_{m}\right)$ represent range-slow time domain. K is the number of scattering centers. $\sigma_{k}\left(t_{m}\right)$, $r_{k}\left(t_{m}\right)$ refer to the backscattering coefficient of the kth scattering center and its instantaneous range to radar. $T_{p}$, B, $r_{ref}$, $f_{c}$ are the pulse width, bandwidth, reference distance, and carrier frequency of the radar signal, respectively.

Taking the translational motion of target into consideration, the motions of scattering centers on a target can be expressed as follows:

(2) $$r_{k}\left(t_{m}\right)=r_{T}\left(t_{m}\right)+r_{0}+R_{k}\left(t_{m}\right),\;k=1,2,\cdots ,K,\;R_{k}\left(t_{m}\right)=A_{k}\sin\left(2\pi f_{M}t_{m}+\varphi_{k}\right),\;k=1,2,\cdots ,K$$

where $r_{T}\left(t_{m}\right)$ is the range induced by translational motion, which is the same for all scatters. $r_{0}$ is the initial relative range between radar and rotating center; $R_{k}\left(t_{m}\right)$ is the range induced by rotational motion of the kth scattering center and $A_{k}$, $\varphi_{k}$, $f_{M}$ are the amplitude, initial phase, and frequency of rotational motion, respectively.

Due to the reference distance measured by narrowband radar, thus the reference distance satisfied

(3) $$r_{ref}\left(t_{m}\right)=r_{0}+r_{T}\left(t_{m}\right)+v\left(t_{m}\right)$$

where $v\left(t_{m}\right)$ denotes the ranging errors between actual reference and measured reference. Due to the attitude of target changes all the time, the ranging errors have randomness.

In Equation (2), the translational motion $r_{T}\left(t_{m}\right)$ is unnecessary and would be removed in m-R signatures by measured reference.

## 3. Estimate Method

In this section, we propose a HRRP-based method to compensate the error of measured reference distance, and estimate the rotational parameters including period, amplitude, and phase. It should be noted that the proposed method is applicable to estimate the parameters that are produced by any micro-motion (not limited to the rotational motion). The proposed method is composed of two stages: (i) calculation for the modified first conditional moment of the range profiles (MFCMRP) and (ii) parameter estimation and image reconstruction.

### 3.1. Calculation for MFCMRP

Compared with function sinc(⋅) used in [6], $\sin c^{2}\left(\cdot \right)$ has smaller sidelobes, so that it is closer to impulse function δ(⋅). On the other hand, the second-order sinc function is more robust than the higher-order ones with respect to the presence of noise. Therefore, the MFCMRP is defined and simplified as follows, and the detailed deduction can be seen in Appendix A.

(4) $$r_{\mathrm{MFCMRP}}\left(t_{m}\right)=-\frac{\int r{\left|H\left(r,t_{m}\right)\right|}^{2}dr}{\int {\left|H\left(r,t_{m}\right)\right|}^{2}dr}\approx r_{T}\left(t_{m}\right)+r_{0}-r_{ref}\left(t_{m}\right)+\frac{\sum_{k=1}^{K}\sigma_{k}^{2}\left(t_{m}\right)\cdot A_{k}\sin\left(2\pi f_{M}t_{m}+\varphi_{k}\right)}{\sum_{k=1}^{K}\sigma_{k}^{2}\left(t_{m}\right)}$$

According to Equation (1), Equation (4) would be simplified as

(5) $$r_{\mathrm{MFCMRP}}\left(t_{m}\right)\approx -v\left(t_{m}\right)+\frac{\sum_{k=1}^{K}\sigma_{k}^{2}\left(t_{m}\right)\cdot A_{k}\sin\left(2\pi f_{M}t_{m}+\varphi_{k}\right)}{\sum_{k=1}^{K}\sigma_{k}^{2}\left(t_{m}\right)}$$

where $\sigma \left(t_{m}\right)$ is dependent on the attitude of the target. Since the periodicity of rotational motion dynamics makes the attitude of target change periodically, $\sigma_{k}\left(t_{m}\right)$ has the same period with rotational motion with respect to slow-time.

(6) $$\sigma_{k}\left(t_{m}\;\right)=\sigma_{k}\left(t_{m}+T_{M}\right)$$

where $T_{M}$ is the period of rotational motion. The fourth term in Equation (6) is a periodic function because the all items in it are periodical, that is,

(7) $$G\left(t_{m}\right)=\frac{\sum_{k=1}^{K}\sigma_{k}^{2}\left(t_{m}\right)\cdot A_{k}\sin\left(2\pi f_{M}t_{m}+\varphi_{k}\right)}{\sum_{k=1}^{K}\sigma_{k}^{2}\left(t_{m}\right)}=G\left(t_{m}+T_{M}\right)$$

From Equations (4)–(7), it is obvious that the MFCMRP can be divided into four terms: translational motion, the initial distance between rotating center and radar, the reference distance, and the periodic term.

### 3.2. Parameters Estimation and Image Reconstruction

When the reference distance is measured by narrowband radar in real-time, as a result of ranging errors, there exist random shifts (residual translational motion) of envelopes of range profile between adjacent pulses. In this situation, the measured reference distance is modeled as a sum of translational motion, random ranging errors, and the the initial relative range between radar and rotating center, as depicted in Equation (3). Moreover, according to Equations (5) and (7), the MFCMRP in (4) can be rewritten as

(8) $$r_{\mathrm{MFCMRP}}\left(t_{m}\right)=G\left(t_{m}\right)-v\left(t_{m}\right)$$

**Figure 1:**
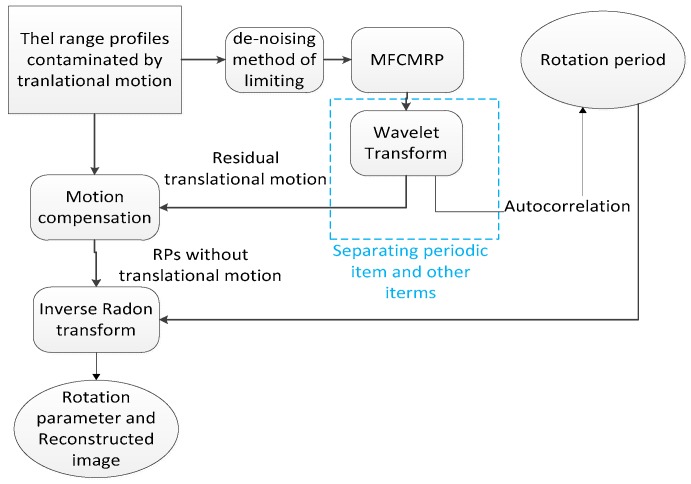
The specific procedures of whole parameter estimated method.

There are two items in the MFCMRP as shown in Equation (8), the former is a periodic item, and the latter is a random item corresponding to ranging errors, also called residual translational motion. For a periodical signal with random noise, the WT is a popular method in stable signal denoising [14,15]. Hence, WT is utilized to separate the periodic item from random item in this paper. Performing autocorrelation on the estimated periodic item would obtain the period estimation. What’s more, the random item is corresponding to the shift range of interfered HRRPs in Equation (1). Compensating the contaminated RPs so as to achieve the rotational m-R signatures, and then utilizing the IRT result of compensated signatures, we can obtain the estimation of rotational motion parameter. The flow chart of the whole procedures is shown in Figure 1, and the specific solution procedures are divided into three steps:

- According to the HRRPs by dechirping echo with online reference, the MFCMRP can be obtained through Equations (4) and (8).
- Performing WT on the MFCMRP can get the random item, the residual translational motion, compensating the contaminated HRRPs with the residual translational motion can get the rotational m-R signatures. Autocorrelation operation is a popular method to estimate the period in periodic function and is utilized for the rotational period estimation.
- Performing inverse radon transform on the compensated HRRPs would get the estimation of rotational parameters, including the amplitude and the phase of rotational motion.

## 4. Experiments

### 4.1. Experiment 1

**Figure 2:**
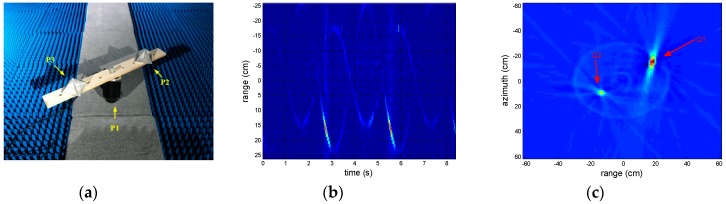
This is an experimental target: (**a**) with the rotating targets, there are two four-side corner reflectors driving by a motor; (**b**) the RPs of the rotating target (SNR = 0 dB); (**c**) the inverse Radon transform of (**b**).

A rotating target without translational motion in our experiment is demonstrated in Figure 2. The target contains two rotating four-side corner reflectors driven by a motor, and the rotational angular is set to 21.4 rpm, the rotational period is 2.8 s, and their rotational amplitudes are 16 and 24 cm, the differential phase of them is π/2rad, respectively. The experiment is carried out in an absorbing chamber, for the transmitted signal, the central frequency $f_{c}=220\;\mathrm{GHz}$, bandwidth B=12.8 GHz, the pulse repetition frequency $f_{s}=1000\;\mathrm{Hz}$ and the power is 1.2 mW. The experiment setup is same as [6], the radar echo viewed as a sum of micro-motion modulation part and translational part. The actual RPs can be seen in Figure 2b, and the IRT of Figure 2b is shown in Figure 2c, where two bright points can be clearly observed. The experimental environment can be seen in [4] except for vibrating interference.

**Figure 3:**
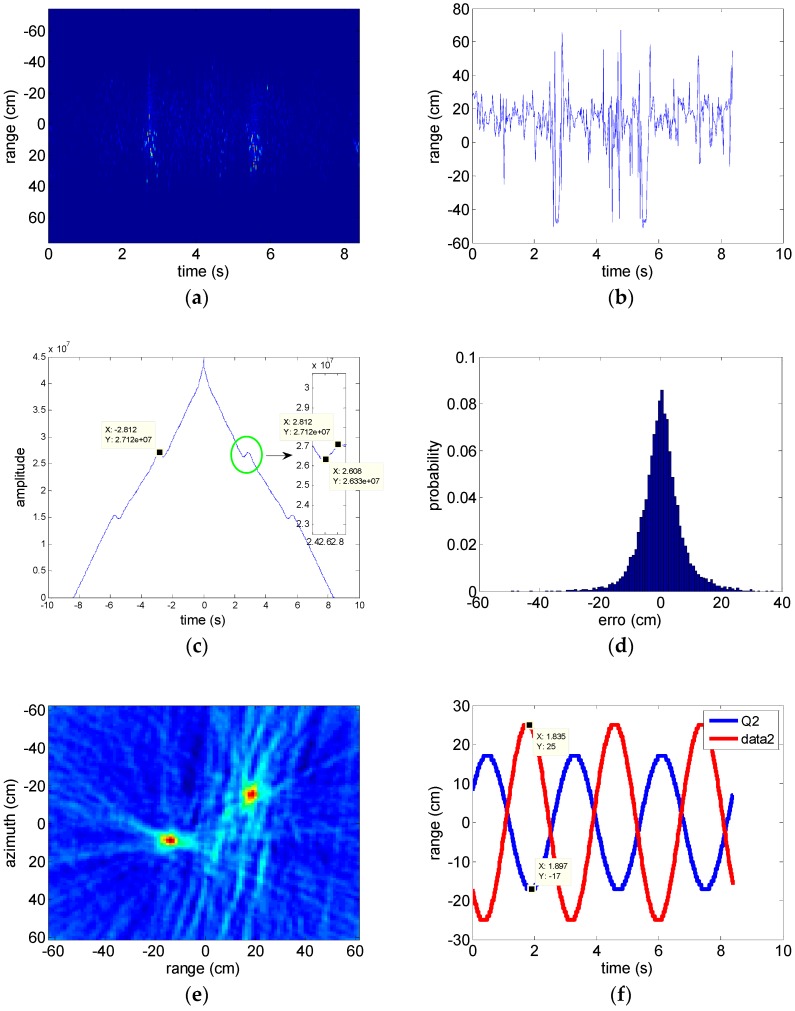
The results of the method proposed in this paper when reference distance is measured. (**a**) RPs contaminated by residual translational motion (SNR = 0 dB); (**b**) the modified first conditional comment of range (MFCMRP) of (**a**); (**c**) the autocorrelation result of (**b**); (**d**) the relationship between translational motion estimation errors and occurrence probabilities; (**e**) inverse radon transform (IRT) result of compensated RPs; (**f**) the parameter of rotational motion estimated by (**e**).

Figure 3a shows the experimental RPs by dechirping on radar echo with a reference distance ranging by narrowband signal, where the RPs contaminated by ranging errors (referred to residual translational motion in this situation). The residual translational motion is modeled as white Gaussian noise with zero mean and variance $\sigma_{v}^{2}=100\;{\mathrm{cm}}^{2}$. The result of performing WT on the MFCMRP is shown in Figure 3b,c including the autocorrelation of the denoised MFCMRP. The rotational period estimation is 2.812 s which agrees well with the actual value. Although the deviation of ranging errors estimation destroy the periodic correlation in the MFCMRP to some extent, the autocorrelation result still expresses a specific point in periodic time interval. The relationship between deviations of translational motion estimated by WT and its occurrence probabilities are shown in Figure 3d, with the majority of deviations ranging from −5 to 5 cm, which contributes to the result of IRT and autocorrelation. Compensating the contaminated RPs with the estimated translational motion and IRT result of the compensated RPs is shown in Figure 3e. According to the positions of peaks in the parameter space, the amplitudes of rotational motion are 25 and 17 cm as shown in Figure 3f. The phases of rotational motion are −28° and 146°, respectively. All of them agree well with the actual value.

**Figure 4:**
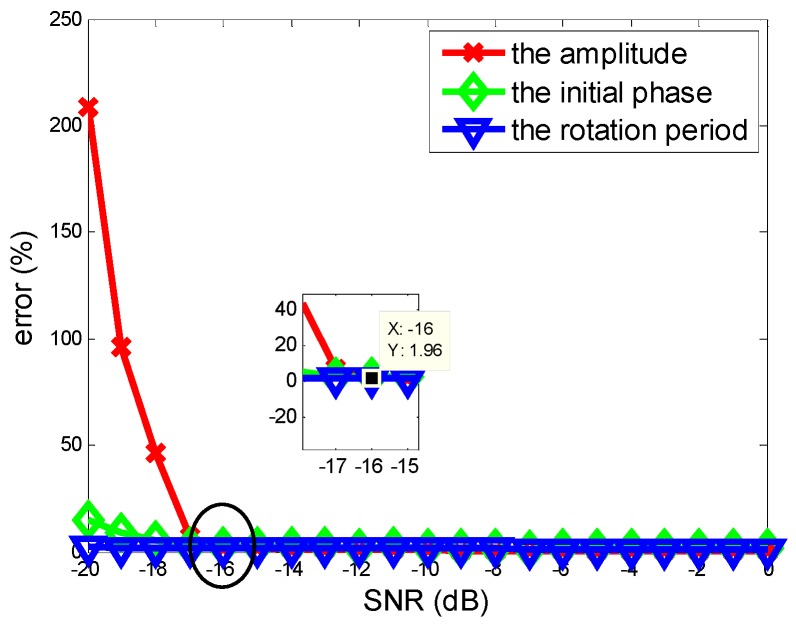
Relationship between signal-to-noise and the parameter estimation errors.

The robustness of the proposed method in this paper under different SNRs is proved in this experiment. The SNR varies from −20 dB to 0 dB with interval 1 dB and Monte Carlo simulations with 100 realizations are run for each SNR. The estimation errors are shown in Figure 4, which is calculated as $error\left(C\right)=\frac{1}{M}\sum_{m=1}^{M}\left|\frac{{\hat{C}}_{m}-C}{C}\right|\times 100\%,C\in \left\{A,f,\varphi \right\}$, where M is the realization time for each SNR, and $C_{m}$ is the estimation of each realization for every parameter.

The simulation target is the corner reflector Q1 in the Figure 2c. As shown in Figure 4, the estimation errors decrease as SNR increase and the rotational amplitude error plays a more important part in the effect of SNR than the initial phase and the period. Due to the benefits of the autocorrelation of the MFCMRP, the rotational period can be estimated well in low SNR. Above all, the method proposed in this paper has a good anti-noise ability, it can also be noted that the estimation error rate for every parameter is no more than 2% when the SNR is greater than −16 dB.

### 4.2. Experiment 2

**Figure 5:**
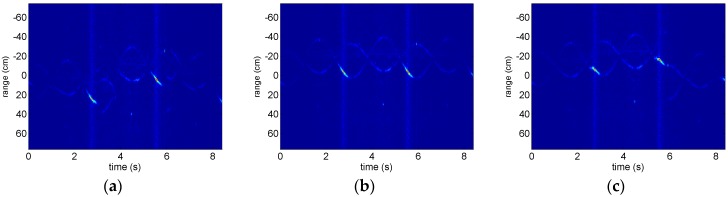
The HRRPs aligned by entropy minimization in different length of smooth window, they are 5, 10, and 15 slow-time sampling units respectively.

**Figure 6:**
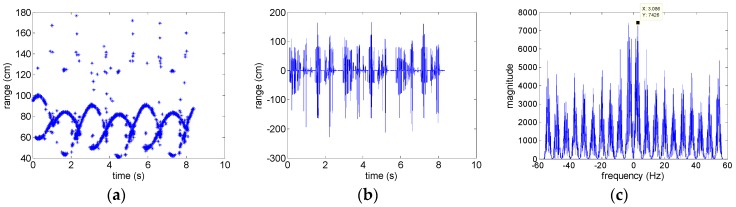
Results motivated by method proposed in [6]. (**a**) instantaneous range of the scattering center with largest energy; (**b**) high-order difference sequence with optimal spectral concentration measure; (**c**) the spectrum of high-order difference sequence.

This experiment demonstrates the result of a method based on high-order difference sequence [6] for comparison. The experimental data is the same with experiment 1. The experimental HRRPs by dechirping on radar echo with the measured reference distance is shown in Figure 3a, and there is not any sinusoidal character compared with Figure 2b because of ranging errors in reference distance (residual translational motion). Motivated by the method based on entropy minimization, the results are different with respect to the different lengths of smooth window. Figure 5a–c denote the range profiles after range alignment corresponding the length of smooth window 5, 10, and 20 slow-time sampling units. It is noted that the optimal length of smooth window would make the right m-R signatures. Thus, the determination for the length of smooth window is vital for range alignment. Selecting 10 slow-time sampling units as the length of smooth window, aligned HRRPs seen in Figure 5b are utilized to estimate the micro-motion period, the procedures of which are depicted in Figure 6. The instantaneous range of the scattering center with largest energy extracted by Viterbi algorithm is plotted in Figure 6a, but that isn’t a sum of sinusoidal signal and polynomial signal described in [6]. The reason is that the relative position of scattering points in rotational motion is front-behind variable. In contrast, the largest energy of scattering center in the processional missile is always the top of missile. The high-order difference sequence of Figure 6a is depicted in Figure 6b, it is hard to recognize sinusoidal character in that. The spectrum of high-order difference sequence is shown in Figure 6c, the estimation of the rotational period is 0.324 s, which disagrees with the actual period. 

From the above results and analysis, the following conclusion would be made: (i) for a micro-motion target, the aligned HRRPs would be affected by the length of smooth window; (ii) if the scattering center of target is a front-behind variable, the instantaneous range of the scattering center with the largest energy isn’t a sum of sinusoidal signal and polynomial signal. Therefore, their applications to estimate micro-motion parameters interfered with translational motion are limited.

## 5. Conclusions

This work proposes a novel HRRP-based method to estimate rotational parameter under the interference of translational motion. The MFCMRP is utilized to describe the rotational dynamics by function approximation, and the relationship between the MFCMRP and translational motion is established. When the reference distance is measured by narrowband radar signal, the MFCMRP contains a periodic item associated with rotational motion and reference distance measured error item.

Taking advantage of WT transform, the ranging error of the reference could be separated to compensate the contaminated HRRPs. Then, performing autocorrelation operation on the periodic item would obtain the rotational period; compensated HRRPs could be utilized to estimate rotational amplitude and initial phase of rotational target by IRT. The results of synthetic and measured radar data shown that the proposed method is effective to estimate the period, amplitude, and phase of micro-motion with high accuracy, and it is convenient for signal processing compared to the method based on high-order difference sequence. In the method motivated by high-order difference sequence, the results of HRRPs range alignment is important for later processing, and the different length smooth window makes different results. However, there is no definite method for length determination. Based on the proposed method, algorithms would be developed to recognize the micro-motion target through estimated amplitude and phase. Further research on how to achieve this would be carried out.

## Appendix A

$$\begin{array}{l} r_{1}\left(t_{m}\right)=\frac{\int r{\left|H\left(r,t_{m}\right)\right|}^{2}dr}{\int {\left|H\left(r,t_{m}\right)\right|}^{2}dr}\approx \frac{\int r\cdot \sum_{k=1}^{K}\sigma_{k}^{2}\left(t_{m}\right)T_{p}^{2}{\left|\sin c\left\{\frac{2B}{c}\left(r-\left(r_{k}\left(t_{m}\right)-r_{ref}\left(t_{m}\right)\right)\right)\right\}\right|}^{2}dr}{{\int \sum_{k=1}^{K}\sigma_{k}\left(t_{m}\right)T_{p}\left|\sin c\left\{\frac{2B}{c}\left(r-\left(r_{k}\left(t_{m}\right)-r_{ref}\right)\right)\right\}\right|}^{2}dr}\\ \approx -\frac{\int r\cdot \sum_{k=1}^{K}\sigma_{k}^{2}\left(t_{m}\right)T_{p}^{2}\cdot \delta \left(r-\left(r_{k}\left(t_{m}\right)-r_{ref}\left(t_{m}\right)\right)\right)dr}{\int \sum_{k=1}^{K}\sigma_{k}^{2}\left(t_{m}\right)T_{p}^{2}\cdot \delta \left(r-\left(r_{k}\left(t_{m}\right)-r_{ref}\left(t_{m}\right)\right)\right)dr}=\frac{\sum_{k=1}^{K}\sigma_{k}^{2}\left(t_{m}\right)T_{p}^{2}\left(r_{k}\left(t_{m}\right)-r_{ref}\left(t_{m}\right)\right)}{T_{p}^{2}\sum_{k=1}^{K}\sigma_{k}^{2}\left(t_{m}\right)}\\ =\frac{\sum_{k=1}^{K}\sigma_{k}^{2}\left(t_{m}\right)T_{p}^{2}\cdot \left(r_{T}\left(t_{m}\right)+r_{0}-r_{ref}\left(t_{m}\right)+R_{k}\left(t_{m}\right)\right)}{T_{p}^{2}\sum_{k=1}^{K}\sigma_{k}^{2}\left(t_{m}\right)}=r_{T}\left(t_{m}\right)+r_{0}+\frac{\sum_{k=1}^{K}\sigma_{k}^{2}\left(t_{m}\right)\cdot A_{k}\sin\left(2\pi f_{M}t_{m}+\varphi_{k}\right)}{\sum_{k=1}^{K}\sigma_{k}^{2}\left(t_{m}\right)}-r_{ref}\left(t_{m}\right) \end{array}$$
